# Accelerated IVIM-corrected DTI in acute hamstring injury: towards a clinically feasible acquisition time

**DOI:** 10.1186/s41747-024-00437-1

**Published:** 2024-03-19

**Authors:** Susanne S. Rauh, Jozef J. M. Suskens, Jithsa R. Monte, Frank Smithuis, Oliver J. Gurney-Champion, Johannes L. Tol, Mario Maas, Aart J. Nederveen, Gustav J. Strijkers, Melissa T. Hooijmans

**Affiliations:** 1 Department of Biomedical Engineering and Physics, Amsterdam University Medical Centers, University of Amsterdam, Amsterdam, The Netherlands; 2 Amsterdam Movement Sciences, Sports, Amsterdam, The Netherlands; 3 Department of Orthopedic Surgery and Sports Medicine, Amsterdam University Medical Centers, University of Amsterdam, Amsterdam, The Netherlands; 4 Department of Radiology and Nuclear Medicine, Amsterdam University Medical Centers, University of Amsterdam, Amsterdam, The Netherlands; 5 Academic Center for Evidence Based Sports Medicine (ACES), Amsterdam, The Netherlands; 6 Amsterdam Collaboration for Health and Safety in Sports (ACHSS), AMC/VUmc IOC Research Center Amsterdam, Amsterdam, The Netherlands

**Keywords:** Athletic injuries, Diffusion tensor imaging, Diffusion magnetic resonance imaging, Muscle (skeletal), Return to sport

## Abstract

**Background:**

Intravoxel incoherent motion (IVIM)-corrected diffusion tensor imaging (DTI) potentially enhances return-to-play (RTP) prediction after hamstring injuries. However, the long scan times hamper clinical implementation. We assessed accelerated IVIM-corrected DTI approaches in acute hamstring injuries and explore the sensitivity of the perfusion fraction (*f*) to acute muscle damage.

**Methods:**

Athletes with acute hamstring injury received DTI scans of both thighs < 7 days after injury and at RTP. For a subset, DTI scans were repeated with multiband (MB) acceleration. Data from standard and MB-accelerated scans were fitted with standard and accelerated IVIM-corrected DTI approach using high *b*-values only. Segmentations of the injury and contralateral healthy muscles were contoured. The fitting methods as well as the standard and MB-accelerated scan were compared using linear regression analysis. For sensitivity to injury, Δ(injured *minus* healthy) DTI parameters between the methods and the differences between injured and healthy muscles were compared (Wilcoxon signed-rank test).

**Results:**

The baseline dataset consisted of 109 athletes (16 with MB acceleration); 64 of them received an RTP scan (8 with MB acceleration). Linear regression of the standard and high-*b* DTI fitting showed excellent agreement. With both fitting methods, standard and MB-accelerated scans were comparable. Δ(injured *minus* healthy) was similar between standard and accelerated methods. For all methods, all IVIM-DTI parameters except *f* were significantly different between injured and healthy muscles.

**Conclusions:**

High-*b* DTI fitting with MB acceleration reduced the scan time from 11:08 to 3:40 min:s while maintaining sensitivity to hamstring injuries; *f* was not different between healthy and injured muscles.

**Relevance statement:**

The accelerated IVIM-corrected DTI protocol, using fewer *b*-values and MB acceleration, reduced the scan time to under 4 min without affecting the sensitivity of the quantitative outcome parameters to hamstring injuries. This allows for routine clinical monitoring of hamstring injuries, which could directly benefit injury treatment and monitoring.

**Key points:**

• Combining high-*b* DTI-fitting and multiband-acceleration dramatically reduced by two thirds the scan time.

• The accelerated IVIM-corrected DTI approaches maintained the sensitivity to hamstring injuries.

• The IVIM-derived perfusion fraction was not sensitive to hamstring injuries.

**Graphical Abstract:**

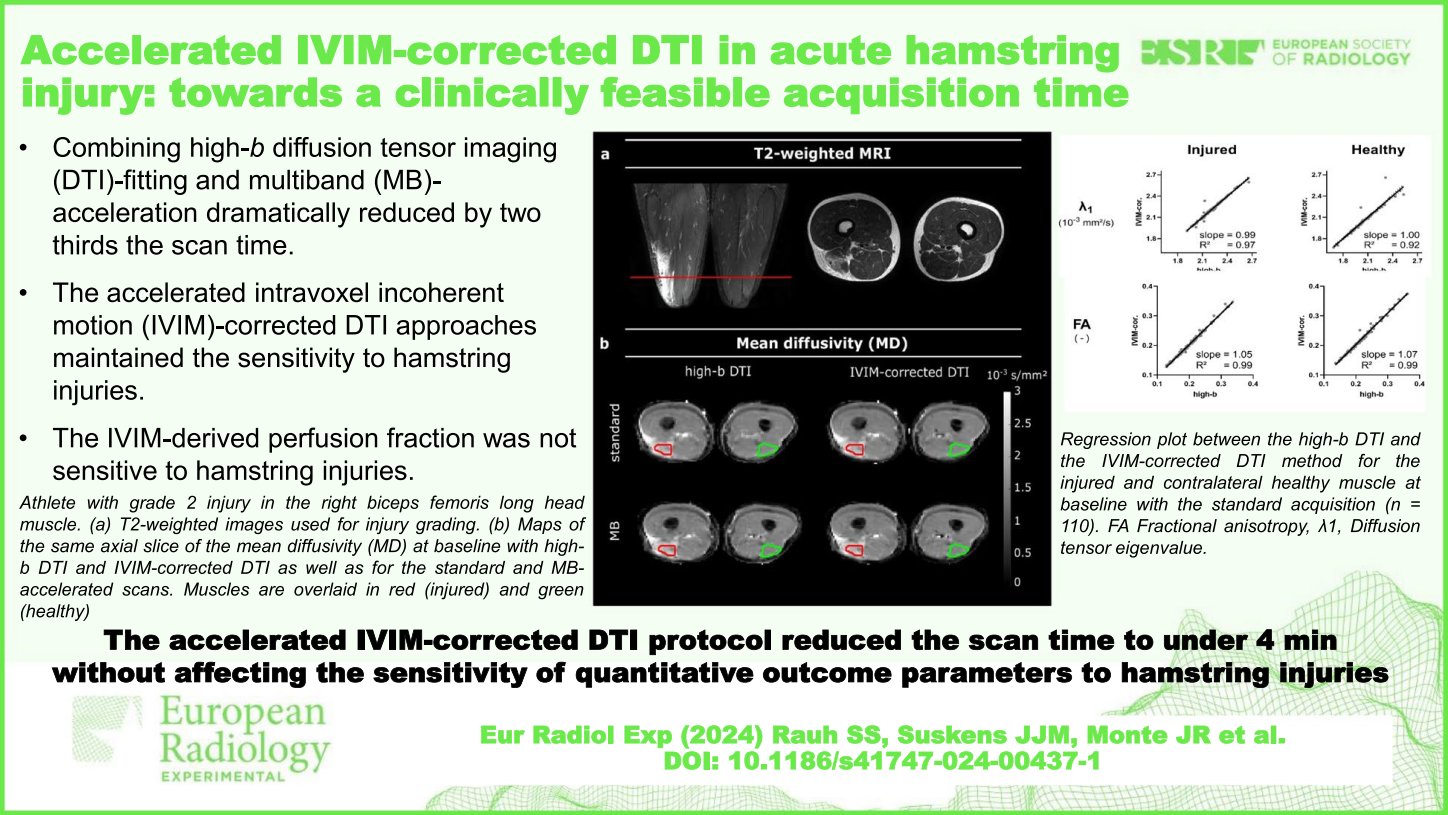

## Background

Acute hamstring injuries are the most common muscle injury in sports involving explosive sprinting [1, 2]. Qualitative magnetic resonance imaging (MRI) techniques, such as T2-weighted imaging, play an important role in the diagnosis of hamstring injuries [3, 4]. However, up to 20% of hamstring injuries show no abnormality on those images [5, 6], and they fail to accurately predict the return-to-play (RTP) time [2, 6–8]. This demonstrates a need for sensitive imaging techniques to monitor injury recovery and improve RTP time predictions.

Diffusion tensor imaging (DTI), a quantitative MRI technique, provides information on the microscopic displacement (diffusion) of water molecules in tissues. Commonly observed microstructural changes in injured muscles include cell swelling, loss of membrane integrity, inflammation, and fibrosis [9]. These affect the water mobility in the tissue, which can be detected by the DTI metrics [10–12]. DTI can depict microstructural changes in skeletal muscle that remain undetected with qualitative MRI techniques [13, 14]. This feature makes DTI a promising candidate to improve RTP time prediction after an acute hamstring injury. At low diffusion weightings (low *b*-values), the (capillary) perfusion causes an additional attenuation of the signal, which leads to a bias in the DTI parameter estimates [15]. Perfusion can be accounted for with intravoxel incoherent motion (IVIM) modeling [16], which separates the signal attenuation into a perfusion part, characterized by a signal perfusion fraction *f* and pseudo-diffusion coefficient D*, and a diffusion part with diffusion coefficient/tensor D. IVIM has proven responsive to inflammation [17] and exercise effects [18] and might be a sensitive parameter to monitor injury recovery. Furthermore, IVIM correction increases the repeatability of DTI parameter estimates in muscles [19].

A limitation of IVIM correction is its requirement for multiple diffusion-sensitizing gradient directions and *b*-values. This requirement results in much longer scan times compared to simple DTI, usually acquired with only two *b*-values, and limits its application for clinical scanning. We have recently shown that IVIM-corrected DTI is suitable to monitor hamstring injury recovery [12]. However, covering the hamstrings requires a large field of view and many slices (typically 30–40). While simple DTI acquisitions of the thighs take typically around 5 min, IVIM-corrected DTI can last up to 11 min [19]. Combined with the anatomical scans, needed to define the injury region and segmentations, the overall scan time increases to 20–30 min, and IVIM-corrected DTI takes up to 50% of the protocol time. This is a burden for the patient. A reduction of the scan time is crucial to further investigate the potential and the clinical relevance of IVIM-corrected DTI for RTP time prediction in clinical studies.

Acceleration of IVIM-corrected DTI can be achieved by acquiring only *b*-values > 200 s/mm^2^, for which the IVIM effect is negligible [15], and/or by acquiring slices simultaneously using a multiband (MB) technique [20]. The aim of this study was to accelerate IVIM-corrected DTI for hamstring muscles to less than four minutes of scan time using the abovementioned two approaches while maintaining sensitivity to hamstring injuries, which would allow for routine clinical monitoring of hamstring injuries. Additionally, we investigated the sensitivity of the IVIM-derived perfusion fraction to hamstring injury.

## Methods

This prospective study was approved by the medical ethics committee of Amsterdam University Medical Centers (NL55671.018.16). All participants provided written informed consent. Part of the study population (*n* = 41) was used in a previous publication [12].

### Subjects

Professional and amateur athletes with clinically confirmed acute hamstring injuries were recruited from 2016 to 2022. Inclusion criteria were as follows: new hamstring injury (< 7 days old) and athlete at least 16 years old. The athletes underwent an MRI scan at two time points: within 7 days after the hamstring injury (baseline) and within 10 days after they returned to full training (RTP). Exclusion criteria for this study were (i) MRI contraindication and (ii) hamstring injury caused by an extrinsic trauma. Participants were excluded from further participation in the following cases: complete proximal tendon avulsion on MRI, injury not in the hamstring muscles, injury not within the field of view of the DTI scan (field of view mismatch), reinjury within 2 months after RTP, other injuries hampering hamstring injury rehabilitation, and insufficient signal-to-noise ratio (SNR) of the DTI data at *b* = 0 s/mm^2^ (SNR < 20) [21].

### MRI protocol

MRI scans of both thighs were acquired in a supine position with a 3-T MRI (Ingenia, Philips, Best, The Netherlands) using a 16-channel anterior receive coil in combination with a 10-channel posterior receive coil. The MRI protocol included an axial T2-weighted turbo spin-echo scan and a coronal T2-weighted Dixon scan for determination of the injury location and injury grading, a proton density-weighted three-dimensional Dixon scan as anatomical reference for segmentations, and a spin-echo echo-planar imaging IVIM-DTI scan. Detailed scan parameters can be found in Table 1. For a subset of participants, the IVIM-DTI scans at baseline and RTP were repeated with MB factor 2. In the following, these two scans are referred to as “standard” and “MB-accelerated.”

**Table 1:** Acquisition parameters

Sequence/parameters	T2-weighted turbo spin-echo	T2-weighted Dixon	Proton density-weighted 3D Dixon	IVIM-DTI	IVIM-DTI with MB factor 2
FOV (mm^3^) (AP × RL × FH)	250.5 × 450 × 237.5	120 × 450 × 450	252 × 480 × 200	252 × 480 × 200	252 × 480 × 210
Voxel size (mm^3^)	0.375 × 0.375 × 2.5	4 × 0.39 × 0.39	1.5 × 1.5 × 2.5	3 × 3 × 5	3 × 3 × 5
Slices	2 × 95	30	80	40	42
Slice orientation	Axial	Coronal	Axial	Axial	Axial
Turbo factor	15	14	–	–	–
Repetition time (ms)	4,969	2,921	8.1	5,914	3,365
Echo time (ms)	70	60	1.35 (Δ TE 1.1)	55	60
Flip angle	90	90	3	90	90
Bandwidth (Hz)	224	245.9	1,430.8	3,064.3	3,058
*b*-value (s/mm^2^) (gradient directions)	–	–	–	0 (8 ×), 5 (3 ×), 10 (3 ×), 20 (3 ×), 50 (3 ×), 100 (3 ×), 200 (10 ×), 400 (10 ×), 600 (12 ×)	0 (8 ×), 5 (3 ×), 10 (3 ×), 20 (3 ×), 50 (3 ×), 100 (3 ×), 200 (10 ×), 400 (10 ×), 600 (12 ×)
Fat suppression	–	–	–	SPAIR + GRFS	SPAIR + GRFS
SENSE factor	2	2.8	3 (RL) / 2 (FH)	1.5	1.5
Partial Fourier	–	–	–	0.8	0.8
Number of excitations	1	1.3	3	2	2
Scan time (min:s)	2 × 4:18	4:40	1:53	11:08 6:27^a^	6:20 3:40^a^

The T2-weighted turbo spin-echo scan covered the whole upper legs, scanned in two stacks. The coronal T2-weighted Dixon scan was planned to cover the hamstring muscles only. Proton density-weighted 3D Dixon and IVIM-DTI were planned to cover the hamstring injury as identified on the T2-weighted turbo spin echo scans. For the multiband accelerated scan, 42 slices were scanned to ensure #slices/MB factor = uneven to avoid slice crosstalk. The total acquisition time for athletes without MB IVIM-DTI scan was 26:17 min:s and for athletes with MB IVIM-DTI scan 32:37 min:s

*3D* Three-dimensional, *GRFS* Gradient reversal fat suppression, *IVIM-DTI* Intravoxel incoherent motion diffusion tensor imaging, *MB* Multiband, *SENSE* Sensitivity encoding, *SPAIR* Spectral adiabatic inversion-recovery

^a^With the omission of low *b*-values

### Acceleration approaches and IVIM-DTI fitting

IVIM-DTI data was denoised and registered to the scanner-reconstructed Dixon water images using QMRITools [22]. The SNR of the *b* = 0 s/mm^2^ diffusion data was calculated based on the noise maps estimated from the denoising as a quality measure [12]. The diffusion tensor with IVIM correction was calculated using nonlinear least-squares fitting in MATLAB (R2021a, The MathWorks, Natick, CA). For both, the standard and MB-accelerated data, the fitting was performed using two approaches:

1. IVIM-corrected DTI [14, 19, 23] (“full fit”)
  - IVIM fit to the mean diffusion-weighted data of both legs
  - Voxel-wise IVIM fit with pseudo-diffusion coefficient D* fixed to the value obtained from A
  - Subtraction of the IVIM component from the full signal
    $${\mathrm S}_\text{DTI}=\mathrm S-{\mathrm S}_{0,\mathrm{IVIM}}\cdot f\cdot\mathrm e^{-\mathrm b\cdot\mathrm D^\ast},$$
    with S being the full signal, S_0,IVIM_ the IVIM signal at *b* = 0 s/mm^2^, *f* the perfusion fraction, and D* the pseudo-diffusion coefficient
  - DTI fit using all *b*-values and diffusion-gradient directions to the remaining signal ${{\text{S}}}_{{\text{DTI}}}$ 

2. High-*b* DTI
  - Data were retrospectively undersampled by discarding *b*-values < 200 s/mm^2^, resulting in *b* = 200, 400, 600 s/mm^2^, and 32 unique diffusion-gradient directions. Since the perfusion contribution to the signal is negligible for *b* ≥ 200 s/mm^2^, the data is now inherently IVIM-corrected
  - Subsequently, the diffusion tensor was fitted to the undersampled data

### Outcome measures

A musculoskeletal radiologist with 5 years of experience (F.S.) identified the slice with the primary location of the injury on the axial and coronal T2-weighted MRI. Subsequently, segmentations were drawn manually in the injured muscle in 14 axial slices covering this primary injury location, using the scanner-reconstructed Dixon out-of-phase images and ITK-snap, version 3.8.0 [24] (www.itksnap.org). In those 14 axial slices, the full muscle area of the injured muscle was segmented. In the contralateral muscle, the matching slices were found in the axial T2-weighted images, and the corresponding control muscle areas were segmented on the Dixon out-of-phase images. Segmentations were performed by two observers (J.M. and S.R.) with 6 and 4 years of experience in musculoskeletal MRI, respectively. In case of no visible injury on the T2-weighted MRI (Peetrons’ grade 0 injury: no abnormality on MRI), the muscle was segmented in all slices using the Dixon out-of-phase data. The segmentations were resized to match the DTI data. Subsequently, the segmentation-averaged values for the mean diffusivity (MD), fractional anisotropy (FA), diffusion tensor eigenvalues *λ*_1_, *λ*_2_, *λ*_3_, and *f* (only from IVIM-corrected DTI) were calculated. As primary outcome measures, we compared the different acceleration methods and their sensitivities to hamstring injuries with the standard scan. As a measure of sensitivity to injury, the difference between injured and contralateral muscle, *i.e.,* Δ(injured-healthy), per parameter, patient, as well as per scan and fitting method was calculated at baseline and RTP. The comparison of the IVIM-DTI parameters between injured and healthy muscles served as a secondary outcome measure.

### Statistical analysis

To compare the standard and MB-accelerated scan and the two fitting methods, linear regression analysis was performed for all IVIM-DTI parameters at baseline (GraphPad Prism 9.5.1, San Diego, CA, USA). We considered a slope between 0.9 and 1.1 and a goodness-of-fit (*R*^2^) > 0.9 as excellent agreement, 0.8–0.9 < slope < 1.1–1.2 and *R*^2^ > 0.7 as good agreement, and 0.7–0.8 < slope < 1.2–1.3 and *R*^2^ > 0.5 as moderate agreement. A Wilcoxon signed-rank test was used to assess differences in sensitivity (Δ(injured-healthy)) between the fitting methods and MB acceleration for all IVIM-DTI parameters at baseline and RTP (SPSS Statistics 28.01.1.1, IBM Corp., Armonk, NY, USA). Differences in IVIM-DTI parameters between the injured and non-injured muscles were compared using a Wilcoxon signed-rank test in SPSS. For all analyses, the threshold for significance was set to 0.05.

## Results

### Athlete inclusion

**Fig. 1:**
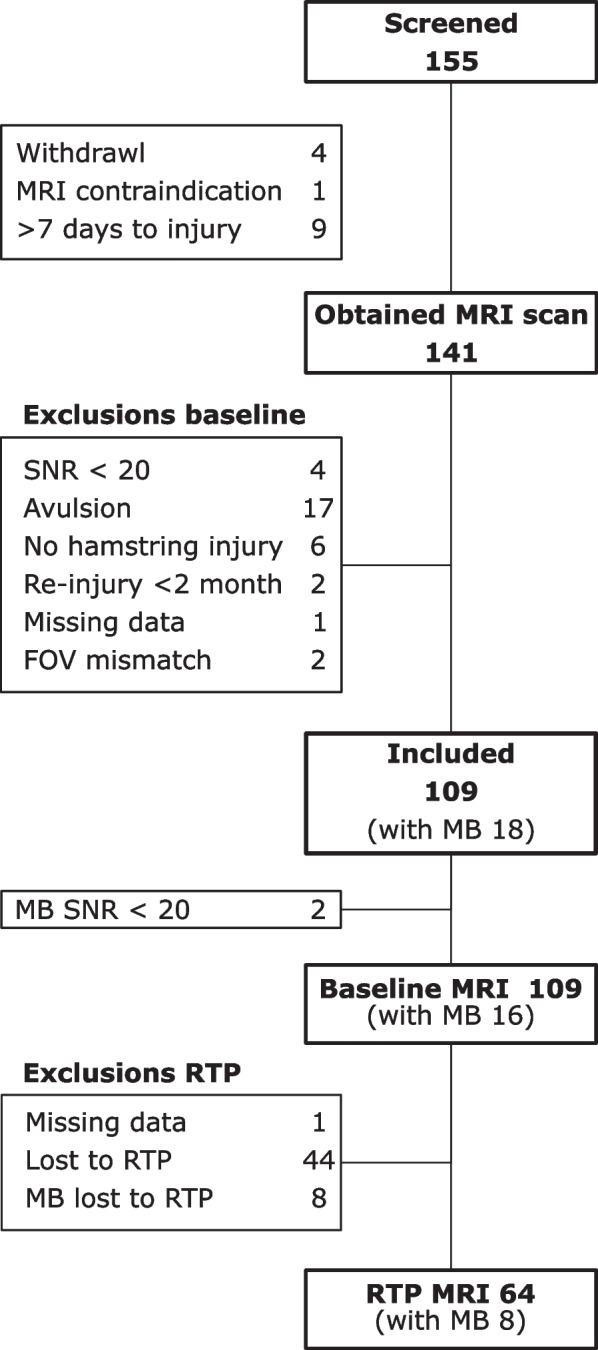
Flow chart of the athlete participation throughout the study. *FOV*, Field of view; *MB*, Multiband; *MRI*, Magnetic resonance imaging; *RTP*, Return-to-play; *SNR*, Signal-to-noise ratio

Details about the athlete participation throughout the study are given in Fig. 1. Initially, 155 athletes were screened for eligibility. Fourteen athletes were excluded because they either chose to withdraw (*n* = 4), had an MRI contraindication (*n* = 1), or had an injury older than 7 days (*n* = 9), resulting in 141 athletes receiving a baseline MRI scan. Due to various criteria (Fig. 1), 32 additional athletes were excluded based on the first MRI scan, leaving 109 athletes (5 female, 104 male, mean age 25.4 years, range 16–53 years) at baseline (Table 2). Eighteen athletes received an MB-accelerated scan at baseline. Two MB-accelerated scans were excluded due to low SNR, resulting in 16 MB-accelerated scans at baseline. At RTP time, 44 athletes chose to withdraw from the study. Sixty-five athletes received an MRI scan at RTP. One athlete was excluded due to experimental problems (missing data), leaving 64 athletes at the RTP time point, of which 8 also received a successful MB-accelerated scan.

**Table 2:** Athlete characteristics

a		**# Included athletes**		**Age (years)**	
	**Male**	104		25.4 ± 7.9	
	**Female**	5		25.2 ± 2.2	
	**Total**	109		25.4 ± 7.8	
b	**Injury grade**	**Number of injuries (percentage)**		**RTP time (days) (number of injuries)**	
	0	7	(6.4%)	9.0 ± 6.5	(5)
	1	22	(20.0%)	28.2 ± 19.5	(17)
	2	81	(73.6%)	50.9 ± 32.4	(65)
	**Total**	110		44.1 ± 31.8	(87)

Characteristics of the included athletes with acute hamstring injury (a) and injury figures (b) including injury grading based on a modified Peetrons’ classification, number (and percentage) of injuries per grade, and return-to-play times for the athletes where RTP time was known. For 23 injuries, the RTP times were not known. The definition of the modified Peetrons’ classification was as follows: grade 0 = no abnormality; grade 1 = edema without architectural distortion; grade 2 = edema with architectural disruption; grade 3 = complete tear (exclusion criteria in this study). Data for age and RTP are given as mean ± standard deviation

*RTP* Return-to-play

### Injury distribution

At baseline, 109 athletes presented with 110 acute hamstring injuries. Of those, 45 injuries were in the left and 65 in the right leg. The biceps femoris long head muscle was injured most often (*n* = 67, 60.9%), followed by the semimembranosus (*n* = 22, 20.0%). In 20 injuries, both the biceps femoris long head and semitendinosus muscles were affected (18.2%), and in 1 injury the biceps femoris long head and the biceps femoris short head muscles were affected (0.9%). The injury Peetrons’ grades and RTP times are listed in Table 2.

### MRI

**Fig. 2:**
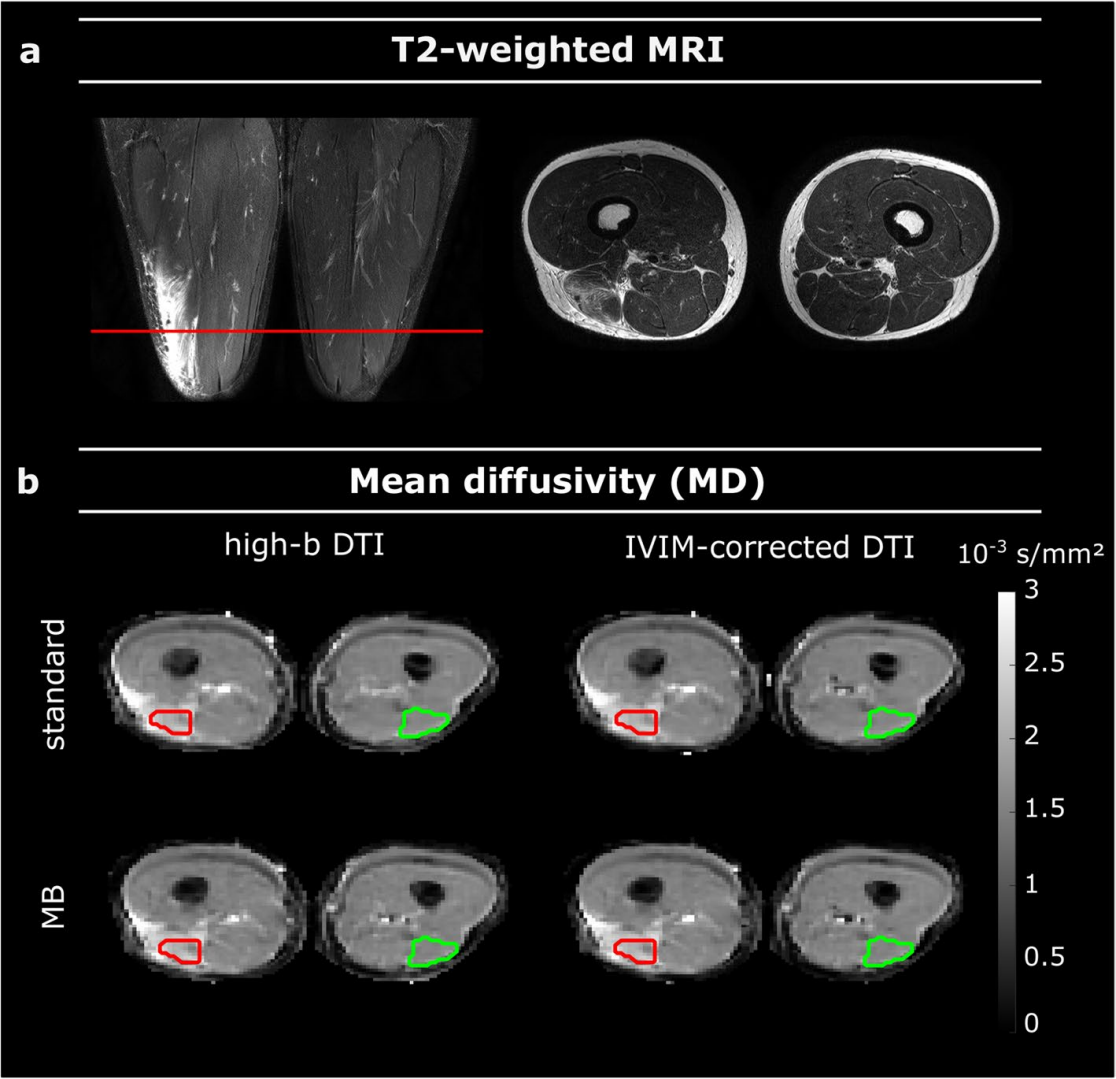
Representative images of an athlete with grade 2 injury in the right biceps femoris long head muscle. **a** T2-weighted images used for injury grading. The red line indicates the slice identified by a radiologist as containing the primary location of the injury. The axial image of this slice is shown. **b** Parameter maps of the same axial slice of the MD at baseline with both fitting methods (high-*b* DTI and IVIM-corrected DTI) as well as for the standard and MB-accelerated scans. The segmentations of the injured and healthy muscles are overlaid in red and green, respectively. *MD*, Mean diffusivity; *MB*, Multiband

Omission of the low *b*-value acquisitions for high-*b* DTI fitting resulted in a 42% scan time reduction compared to the fully sampled protocol (absolute scan time 6:27 *versus* 11:08 min:s). MB acceleration with a factor of 2 reduced the scan time by almost 50% (6:20 *versus* 11:08 min:s). MB combined with the omission of low *b*-values yielded a scan time reduction of 67% as compared to the standard scan (3:40 *versus* 11:08 min:s). The mean SNR values with the standard scan for the injured/healthy muscles were 68/45 at baseline and 52/45 at RTP, respectively. With MB acceleration, SNR in the injured/healthy muscles was 67/38 at baseline and 42/35 at RTP. Figure 2 shows representative T2-weighted images and quantitative MD maps with the four different approaches (standard acquisition with high-*b* DTI fit and full IVIM-corrected DTI fit, and MB-accelerated acquisition with high-*b* DTI fit and full IVIM-corrected DTI fit) for an athlete with a Peetrons’ grade 2 hamstring injury. Visually, the four MD maps were nearly indistinguishable, which will be quantified for the various outcome measures further on. MD values of the injured muscle were elevated and visible as hyperintense areas on the MD maps.

### Primary outcome measure: correlation between methods

**Fig. 3:**
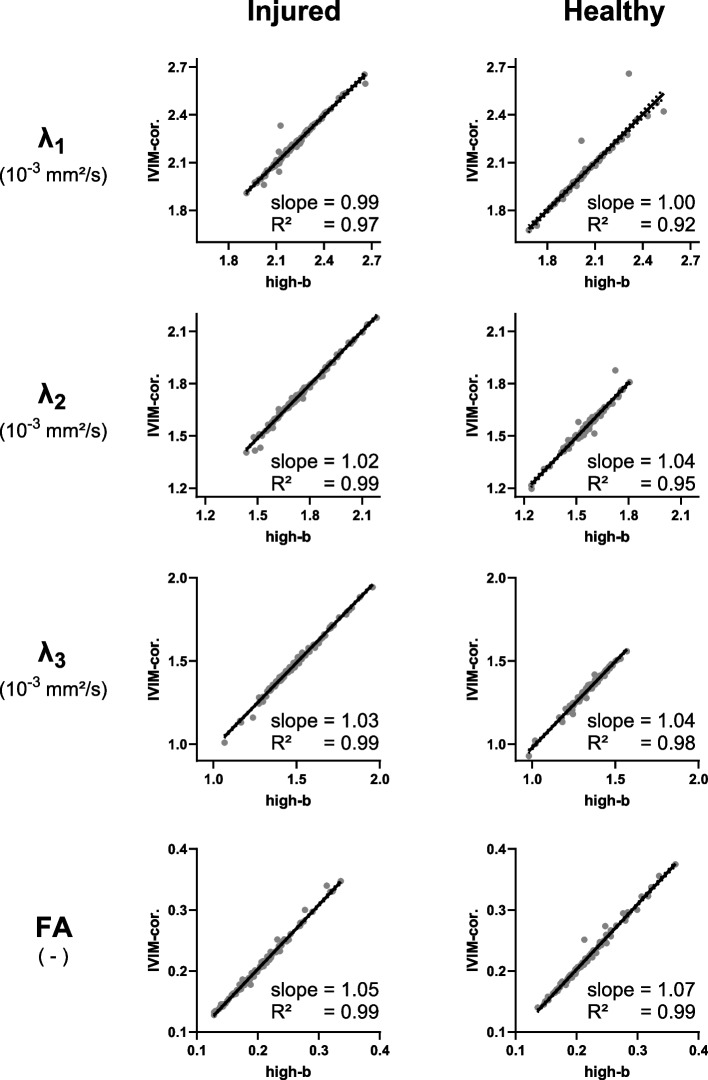
Regression plot and 95% confidence intervals (dotted lines) between the high-*b* DTI and the full fit (IVIM-corrected DTI) method for the injured and contralateral healthy muscle at baseline with the standard acquisition (*n* = 110). The slope and *R*^2^ values are reported for each parameter. *DTI,* Diffusion tensor imaging; *FA*, Fractional anisotropy; *IVIM*, Intravoxel incoherent motion; *λ*_*1*_*, λ*_*2*_*, λ*_*3*_, diffusion tensor eigenvalues

**Fig. 4:**
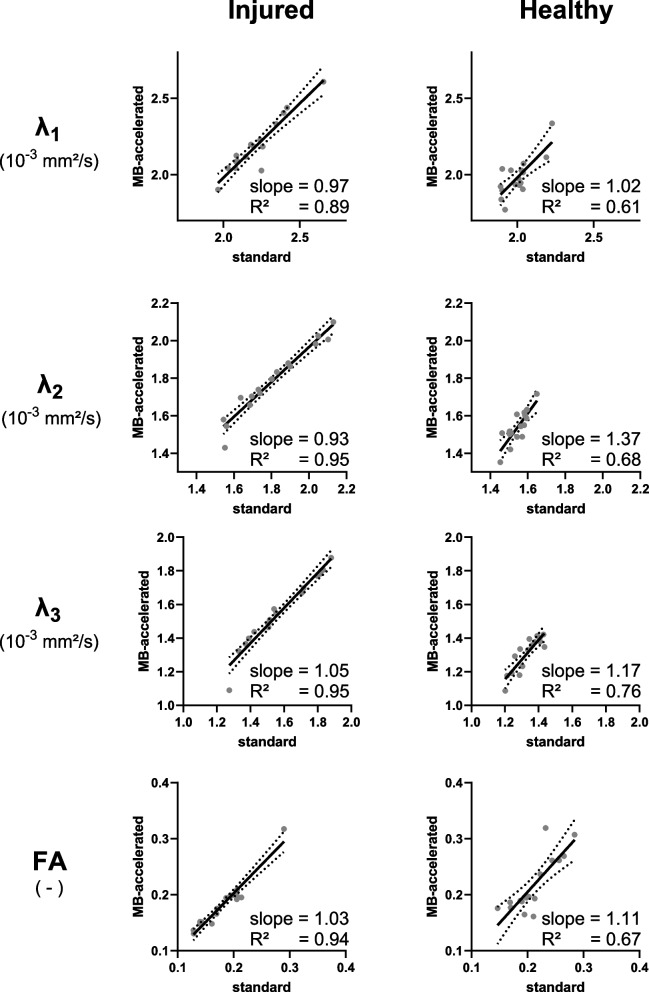
Regression analysis between standard and MB-accelerated scan at baseline (*n* = 16) for the IVIM-DTI parameters obtained with the high-*b* DTI fitting method. The slope and *R*^*2*^ values are reported for each parameter. The 95% confidence intervals are given by the dotted lines. *DTI*, Diffusion tensor imaging; *FA*, Fractional anisotropy; *IVIM*, Intravoxel incoherent motion; *λ*_*1*_*, λ*_*2*_*, λ*_*3*_, diffusion tensor eigenvalues; *MB*, Multiband

**Fig. 5:**
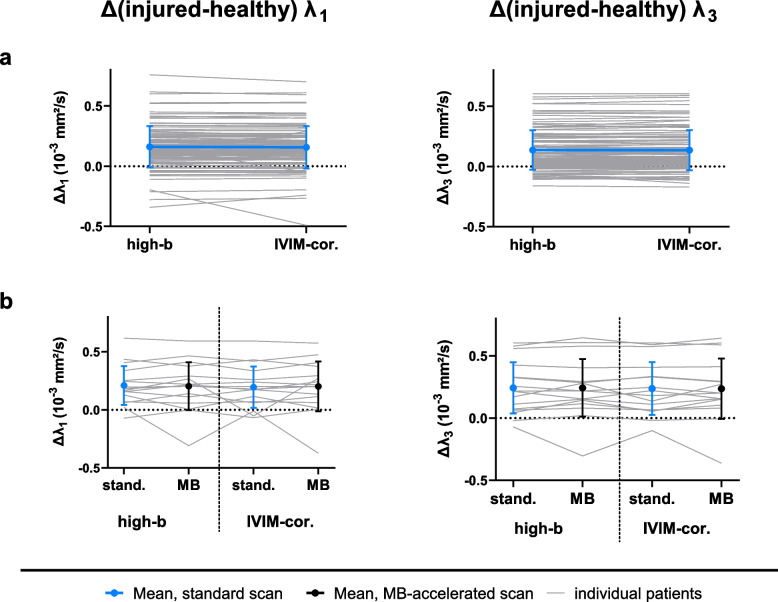
Difference between injured and healthy contralateral muscle (Δ) as a measure of sensitivity for the diffusion tensor eigenvalues λ_1_ and λ_3_ for both, standard acquisition (**a**) and MB-accelerated scans (**b**). Data is shown for the high *b*-value accelerated fitting method as well as for the IVIM-corrected standard fit. Blue and black solid circles indicate group mean ± standard deviation, whereas the grey lines are the data of individual athletes.* λ*_*1*_*, λ*_*3*_, diffusion tensor eigenvalues; *IVIM*, Intravoxel incoherent motion; *MB*, Multiband

Table 3 gives an overview of the outcome measures in the injured and contralateral muscles with the standard acquisition, for both fitting methods, and for all subjects at baseline and RTP, and Table 4 for the subset of athletes who received a standard and MB-accelerated scan. Comparing the two fitting methods in the standard scan at baseline (*n* = 110), we found an excellent agreement for all parameters for both, the injured and contralateral healthy muscles, with the slope of the linear regression being 0.99–1.07 (mean 1.03) and *R*^2^ = 0.92–0.99 (mean 0.97). This is reflected in Fig. 3 and Table 3. Comparing the IVIM-DTI parameters obtained from the MB-accelerated with those from the standard scan for both fitting methods, for the high-*b* DTI fit, the slope of the linear regression was in the range of 0.93–1.37 (mean 1.10) and *R*^2^ 0.61–0.95 (mean 0.81, Fig. 4), and for the full IVIM-corrected DTI fit, we found a slope of 0.67–1.31 (mean 1.02) and *R*^2^ of 0.36–0.94 (mean 0.74). As can be observed in Fig. 4, the agreement between MB acceleration and standard scan was better in the injured muscle than in the contralateral healthy muscle. The moderate to good agreement found between standard and MB-accelerated scans is reflected by slightly lower DTI metrics with MB acceleration (Table 4). The sensitivity to hamstring injuries, measured as Δ(injured-healthy), was not significantly different between the high-*b* DTI and full IVIM-corrected DTI fitting methods, with *p*-values at baseline/RTP: *λ*_1_ = 0.274/0.068, *λ*_2_ = 0.651/0.478, *λ*_3_ = 0.406/0.363, MD = 0.630/0.168, FA = 0.590/0.621 (Fig. 5a). Similarly, we found no significant difference in sensitivity between standard and MB-accelerated scan with both fitting methods (Fig. 5b), with the *p*-values for high-*b* DTI fitting at baseline/RTP *λ*_1_ = 0.605/1.000, *λ*_2_ = 0.569/0.208, *λ*_3_ = 0.535/0.779, MD = 0.756/0.779, FA = 0.717/0.779 and for full IVIM-corrected DTI fitting at baseline/RTP *λ*_1_ = 0.569/0.889, *λ*_2_ = 0.569/0.674, *λ*_3_ = 0.605/0.674, MD = 0.642/0.674, FA = 1.000/1.000, *f* = 0.301/1.000.

**Table 3:** Diffusion tensor imaging parameters with standard acquisition

		Baseline (*n* = 110)			RTP (*n* = 64)		
		Injured	Healthy	*p*-value	Injured	Healthy	*p*-value
*λ*_1_ (10^−3^ mm^2^/s)	High-*b*	2.19 ± 0.14	2.03 ± 0.14	**< 0.001**	2.09 ± 0.11	2.04 ± 0.15	**< 0.001**
	IVIM-cor	2.18 ± 0.14	2.03 ± 0.15	**< 0.001**	2.09 ± 0.11	2.04 ± 0.14	**< 0.001**
*λ*_2_ (10^−3^ mm^2^/s)	High-*b*	1.73 ± 0.15	1.56 ± 0.09	**< 0.001**	1.61 ± 0.09	1.57 ± 0.09	**0.015**
	IVIM-cor	1.72 ± 0.16	1.55 ± 0.10	**< 0.001**	1.60 ± 0.09	1.57 ± 0.09	**0.022**
*λ*_3_ (10^−3^ mm^2^/s)	High-*b*	1.48 ± 0.15	1.34 ± 0.10	**< 0.001**	1.38 ± 0.10	1.36 ± 0.10	0.302
	IVIM-cor	1.47 ± 0.16	1.34 ± 0.11	**< 0.001**	1.37 ± 0.10	1.36 ± 0.10	0.245
MD (10^−3^ mm^2^/s)	High-*b*	1.80 ± 0.13	1.64 ± 0.09	**< 0.001**	1.69 ± 0.08	1.66 ± 0.09	**0.007**
	IVIM-cor	1.79 ± 0.14	1.64 ± 0.10	**< 0.001**	1.69 ± 0.08	1.65 ± 0.08	**0.006**
Fractional anisotropy	High-*b*	0.20 ± 0.04	0.22 ± 0.04	**0.008**	0.22 ± 0.04	0.21 ± 0.04	0.078
	IVIM-cor	0.21 ± 0.04	0.22 ± 0.05	**0.010**	0.22 ± 0.04	0.21 ± 0.05	0.137
*f*	IVIM-cor	0.06 ± 0.02	0.06 ± 0.02	0.334	0.06 ± 0.02	0.06 ± 0.02	0.774

Group averaged diffusion tensor imaging parameters with the standard acquisition for injured and contralateral healthy muscles at baseline and return-to-play and both fitting methods and the *p*-values of the Wilcoxon signed-rank test, comparing the injured and healthy parameters. Data are mean ± standard deviation of the data acquired with the standard scan without multiband acceleration. Significant *p*-values are indicated in bold

*f* IVIM-derived perfusion fraction, *IVIM* Intravoxel incoherent motion, *IVIM-cor* IVIM-corrected DTI, *λ*_*1*_*, λ*_*2*_*, λ*_*3*_ diffusion tensor eigenvalues, *MD* Mean diffusivity, *RTP* Return-to-play

**Table 4:** Diffusion tensor imaging parameters with multiband-accelerated acquisition

			Baseline (*n* = 16)			RTP (*n* = 8)		
			Injured	Healthy	*p*-value	Injured	Healthy	*p*-value
*λ*_1_ (10^−3^ mm^2^/s)	High-*b*	Standard	2.21 ± 0.17	2.00 ± 0.10	**< 0.001**	2.02 ± 0.08	1.97 ± 0.09	0.237
		Multiband	2.19 ± 0.17	1.98 ± 0.12	**0.008**	2.02 ± 0.08	1.96 ± 0.05	0.160
	IVIM-cor	Standard	2.21 ± 0.17	2.02 ± 0.11	**0.002**	2.02 ± 0.07	1.99 ± 0.10	0.553
		Multiband	2.18 ± 0.18	1.98 ± 0.12	**0.006**	2.02 ± 0.08	1.96 ± 0.05	0.207
*λ*_2_ (10^−3^ mm^2^/s)	High-*b*	Standard	1.80 ± 0.19	1.55 ± 0.05	**< 0.001**	1.58 ± 0.09	1.51 ± 0.10	**0.046**
		Multiband	1.78 ± 0.18	1.54 ± 0.08	**0.002**	1.57 ± 0.09	1.50 ± 0.12	**0.024**
	IVIM-cor	Standard	1.80 ± 0.19	1.55 ± 0.05	**0.001**	1.57 ± 0.09	1.52 ± 0.10	0.173
		Multiband	1.77 ± 0.19	1.54 ± 0.08	**0.004**	1.55 ± 0.10	1.50 ± 0.11	0.116
*λ*_3_ (10^−3^ mm^2^/s)	High-*b*	Standard	1.57 ± 0.19	1.33 ± 0.07	**0.001**	1.36 ± 0.09	1.31 ± 0.13	0.140
		Multiband	1.55 ± 0.20	1.31 ± 0.10	**0.004**	1.31 ± 0.12	1.24 ± 0.19	0.325
	IVIM-cor	Standard	1.56 ± 0.19	1.32 ± 0.08	**0.001**	1.35 ± 0.09	1.31 ± 0.12	0.105
		Multiband	1.53 ± 0.21	1.30 ± 0.10	**0.005**	1.28 ± 0.13	1.23 ± 0.19	0.575
MD (10^−3^ mm^2^/s)	High-*b*	Standard	1.86 ± 0.17	1.63 ± 0.05	**< 0.001**	1.65 ± 0.08	1.60 ± 0.10	0.127
		Multiband	1.84 ± 0.18	1.61 ± 0.08	**0.003**	1.63 ± 0.08	1.57 ± 0.11	**0.030**
	IVIM-cor	Standard	1.86 ± 0.18	1.63 ± 0.06	**< 0.001**	1.65 ± 0.08	1.61 ± 0.10	0.260
		Multiband	1.83 ± 0.19	1.61 ± 0.08	**0.004**	1.62 ± 0.08	1.56 ± 0.11	0.063
Fractional anisotropy	High-b	Standard	0.18 ± 0.04	0.21 ± 0.04	**0.026**	0.21 ± 0.04	0.21 ± 0.04	0.916
		Multiband	0.18 ± 0.04	0.22 ± 0.05	**0.049**	0.23 ± 0.06	0.24 ± 0.07	0.498
	IVIM-cor	Standard	0.18 ± 0.04	0.22 ± 0.04	**0.033**	0.21 ± 0.04	0.22 ± 0.03	0.590
		Multiband	0.19 ± 0.05	0.22 ± 0.05	0.065	0.23 ± 0.06	0.24 ± 0.07	0.497
*f*	IVIM-cor	Standard	0.06 ± 0.01	0.06 ± 0.02	0.822	0.07 ± 0.03	0.05 ± 0.01	0.223
		Multiband	0.06 ± 0.01	0.06 ± 0.02	0.339	0.08 ± 0.04	0.07 ± 0.02	0.416

Group averaged diffusion tensor imaging parameters for the subgroup who received a multiband accelerated scan. Data are mean ± standard deviation of the data acquired with the standard scan and with MB acceleration. The *p*-value of the Wilcoxon signed-rank test, comparing injured and healthy parameters, is shown (significant *p*-values in bold)

*f* IVIM-derived perfusion fraction, *IVIM* Intravoxel incoherent motion, *IVIM-cor* IVIM-corrected DTI, *λ*_*1*_*, λ*_*2*_*, λ*_*3*_ diffusion tensor eigenvalues, *MB* Multiband, *MD* Mean diffusivity, *RTP* Return-to-play

### Secondary outcome measure: injured *versus* healthy muscle

**Fig. 6:**
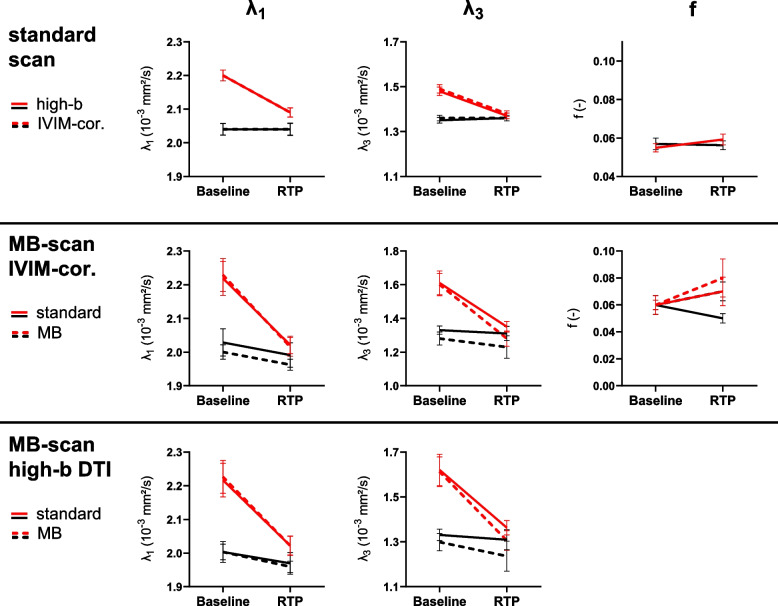
Diffusion tensor eigenvalues *λ*_1_ and *λ*_3_ and IVIM-derived perfusion fraction *f* at baseline and return-to-play for injured (red) and healthy contralateral muscles (black) for both fitting methods. The standard scan (*n* = 64) is shown in the top row, and the standard is compared to the multiband accelerated scan (*n* = 8) in the bottom rows for both fitting methods. Data are shown as group mean ± standard error of the mean. *IVIM*, Intravoxel incoherent motion; *MB*, Multiband; *RTP*, Return-to-play

At baseline with the standard scan, for both fitting methods, all parameters except *f* were significantly different between the injured and healthy muscles (Table 3). The diffusion parameters *λ*_1_, *λ*_2_, *λ*_3_ and MD in the injured muscle were elevated, while FA was lower. The same pattern was seen for the MB-accelerated data (Table 4). At RTP, the differences between the injured and healthy muscles normalized (Fig. 6). However, significant differences between injured and healthy muscles were still observed for *λ*_1_, *λ*_2_ and MD for the standard scans. In the subset of athletes with standard and MB-accelerated scans, a significant difference at RTP was only observed for *λ*_2_ with the high-*b* DTI fitting method with both, standard and MB-accelerated scanning, and for MD with MB acceleration and the high-*b* fitting method, but not for the other parameters.

## Discussion

In this study, we successfully accelerated IVIM-corrected DTI of the hamstrings up to 3:40 min:s scan time, without affecting the sensitivity to hamstring injuries. This acceleration is an important step towards routine clinical usage to monitor hamstring injury recovery and investigate improved RTP time prediction. We prospectively included 109 athletes with acute hamstring injuries to validate our accelerated protocol. The acceleration of the DTI examination was achieved using two approaches. In the first approach, low *b*-value acquisitions were retrospectively omitted, effectively eliminating the effects of perfusion on the signal and permitting a simple DTI fit to the data. The second approach involved the use of MB acceleration, which permitted measuring multiple slices simultaneously, hence reducing scan time. Both approaches to reduce scan time, as well as their combined application, proved feasible. The sensitivity of the quantitative parameters to hamstring injuries, measured as Δ(injured-healthy), was preserved with the accelerated scans, for both, baseline and RTP scans. The IVIM-derived perfusion fraction was not significantly different between injured and healthy muscles.

We found that all IVIM-DTI parameters could depict changes between the injured and healthy muscle at baseline, except for the perfusion fraction. This was not weakened by accelerating the scan with either high-*b* DTI, MB acceleration, or a combination of both. The elevated MD and eigenvalues and lower FA observed in the injured muscle are in line with literature findings [10–14, 25]. As recently suggested by Monte et al. [12], we could confirm that DTI provides sensitive biomarkers for hamstring injury detection and recovery monitoring in a large patient population. We still found differences in some parameters at RTP time. This suggests incomplete healing of the hamstring injuries, which might be associated with increased re-injury risk and needs further investigations in the future.

We also investigated the added value of the perfusion fraction for hamstring injury detection. Our results suggest that *f* is not different in the injured compared to the contralateral healthy hamstring muscle, indicating that *f* is not sensitive to hamstring injury. However, for the parameter estimation, we did not correct for T2 effects and averaged over the diffusion directions to model an isotropic pseudo-diffusion, which could negatively impact the estimation. Incorporating a T2 correction [26] or taking into account the anisotropy of the capillary network in skeletal muscle [27] might be useful in the future to accurately estimate IVIM-DTI parameters. Nevertheless, in the current setting, sampling of low b-values for IVIM parameter estimation seems redundant, and high-*b* DTI can be regarded as the method of choice for hamstring injury assessment.

Comparing the two fitting approaches, we found slopes and *R*^2^ close to one suggesting excellent agreement between the standard (IVIM-corrected DTI) and accelerated (high-*b* DTI) fit for all IVIM-DTI parameters. Comparing the standard and MB-accelerated acquisition approaches, we found moderate-to-good agreement between standard and MB-accelerated scans with both fitting methods. This somewhat lower agreement is likely due to the lower number of subjects since only 16 out of the 109 athletes received an MB-accelerated scan. Investigating MB acceleration in more subjects might strengthen these findings. Interestingly, the agreement in the contralateral healthy muscle was lower than in the injured muscle. This might be related to B_1_ inhomogeneities in the scanner [19]. Another relevant factor could be the lower SNR in the MB-accelerated scans, resulting in a slight underestimation of the DTI metrics. A reason for that might be the slightly higher TE needed with MB acceleration (60 ms compared to 55 ms without MB). However, by comparing the Δ(injured-healthy) values only, we could show that the sensitivity is preserved with MB acceleration. This is in agreement with previously published work which showed that an MB factor of 2 is suitable for skeletal muscle applications and yields an optimal balance between acceleration and image quality [28, 29].

This accelerated protocol, using high-*b* DTI with MB acceleration, can be used in future research to monitor hamstring injury recovery and to investigate the ability of DTI to predict RTP times. This is of clinical relevance as, currently, conventional techniques are lacking for monitoring muscle injury recovery and predicting RTP. In this study, we focused on athletes with hamstring injuries. However, the accelerated high-*b* DTI protocol can directly be applied to investigate muscle injuries in other muscle groups, like the lower legs or the quadriceps. The high-*b* DTI approach can also be beneficial for other applications. For example, DTI has been used to monitor training effects in the hamstrings [30, 31] and to detect disease progression in neuromuscular diseases [32, 33], and using IVIM correction would be beneficial to avoid an IVIM bias in the DTI parameters. Moreover, our accelerated protocol could facilitate the use of DTI to study muscle microstructure and quality, which is an important topic for research, for example in cerebral palsy [34], neuromuscular disorders [35], and aging [36]. Our approach might also be interesting for DTI with longer diffusion times, which is typically achieved by a stimulated echo sequence, and results in a shift of *b* = 0 s/mm^2^ to higher *b*-values due to the contribution from imaging gradients [37]. This causes different IVIM contributions at different diffusion times. The use of our high-*b* DTI approach can eliminate this IVIM effect.

This study had some limitations. First, only 16 athletes received an MB-accelerated IVIM-DTI scan at baseline and 8 athletes at RTP due to small innovations in the scanner capabilities in the end stage of subject inclusion. Our results suggest preserved sensitivity to hamstring injury with MB acceleration; however, a study including more subjects to confirm our findings and ensure preserved data quality is highly desirable. The lower SNR with MB acceleration should be investigated in more depth as sufficient data quality is essential for a reliable IVIM-DTI fit. Second, the pseudo-diffusion coefficient D* was not considered as a clinical outcome parameter due to its high uncertainty in skeletal muscle [38]. Advanced fitting algorithms like machine learning [39, 40] or model-based reconstruction approaches [41] might overcome this challenge in the future. Studying D* might yield additional information about the muscle microstructure and its alterations in hamstring injuries. Third, the segmentations were drawn over a fixed number of slices covering the center of the injury. This caused an averaging effect for injuries in the muscle belly since more healthy tissue will be included in the injured muscle segmentation. Especially for mild injuries (Peetrons’ grades 0 and 1), which typically have a smaller extent, the mean value might average out the injury effect on the DTI parameters. Additional analysis, such as histogram analysis, could overcome this issue [42]. Fourth, for the high-*b* DTI approach, data was undersampled retrospectively. The shorter acquisition time by omitting low *b*-values prospectively might additionally reduce motion artifacts and could thus further improve the data quality. Fifth, only five female athletes were included in our study. While we do not expect differences in the sensitivity of DTI to hamstring injuries in female athletes compared to males, women typically have more subcutaneous fat [43], which could result in incomplete fat suppression and thus influence the DTI metrics. This effect might be more pronounced in the MB-accelerated protocol and should be investigated in more depth in future research.

In conclusion, high-*b* DTI fitting and high-*b* DTI fitting combined with MB acceleration factor 2 reduces the scan time to 6:27 and 3:40 min:s, respectively, while maintaining sensitivity to hamstring injuries. This accelerated protocol allows for routine clinical usage which could directly benefit injury treatment and injury monitoring for athletes.

## Data Availability

The datasets used and analyzed during the current study are available from the corresponding author upon reasonable request.

## Abbreviations

DTI: Diffusion tensor imaging
*f*: IVIM-derived perfusion fraction
FA: Fractional anisotropy
IVIM: Intravoxel incoherent motion
MB: Multiband
MD: Mean diffusivity
MRI: Magnetic resonance imaging
RTP: Return-to-play
SNR: Signal-to-noise ratio
*λ*_1_, * λ*_2_, * λ*_3_: Diffusion tensor eigenvalues
